# Evaluation of Solid Supports for Slide- and Well-Based Recombinant Antibody Microarrays

**DOI:** 10.3390/microarrays5020016

**Published:** 2016-06-08

**Authors:** Anna S. Gerdtsson, Linda Dexlin-Mellby, Payam Delfani, Erica Berglund, Carl A. K. Borrebaeck, Christer Wingren

**Affiliations:** 1 Department of Immunotechnology, Lund University, Medicon Village, Lund SE-22381, Sweden; anna.sandstrom_gerdtsson@immun.lth.se (A.S.G.); linda.dmellby@gmail.com (L.D.-M.); payam.delfani@immun.lth.se (P.D.); erica.berglund@immun.lth.se (E.B.); carl.borrebaeck@immun.lth.se (C.A.K.B.); 2 Clinical Cancer Research using Emerging Advanced Technologies for Health (CREATE), Lund University, Medicon Village, Lund SE-22381, Sweden

**Keywords:** antibody microarrays, affinity proteomics, solid support, array-in-well, source plate, microarray scanning

## Abstract

Antibody microarrays have emerged as an important tool within proteomics, enabling multiplexed protein expression profiling in both health and disease. The design and performance of antibody microarrays and how they are processed are dependent on several factors, of which the interplay between the antibodies and the solid surfaces plays a central role. In this study, we have taken on the first comprehensive view and evaluated the overall impact of solid surfaces on the recombinant antibody microarray design. The results clearly demonstrated the importance of the surface-antibody interaction and showed the effect of the solid supports on the printing process, the array format of planar arrays (slide- and well-based), the assay performance (spot features, reproducibility, specificity and sensitivity) and assay processing (degree of automation). In the end, two high-end recombinant antibody microarray technology platforms were designed, based on slide-based (black polymer) and well-based (clear polymer) arrays, paving the way for future large-scale protein expression profiling efforts.

## 1. Introduction

Affinity proteomics, mainly represented by antibody microarrays, have emerged as an important tool within proteomics, providing unique opportunities for multiplexed protein expression profiling in both health and disease [1,2,3]. In this context, we have developed a recombinant antibody microarray technology platform for protein profiling of crude, directly-labelled proteomes [4,5,6]. The design and performance of antibody (protein) microarrays and how they are processed are dependent on several factors, of which the interplay between the antibodies (proteins) and the solid surfaces plays a pivotal role [5,7,8,9,10,11,12,13]. In more detail, the solid surfaces, including both the source plate to which the antibodies are loaded prior to printing and the surface onto which the antibodies are printed, will have a direct impact on the printing performances, array format (slide and well based), assay performances, assay processing (degree of automation) and sensing (slide- and plate-based scanners) [8,14,15]. Early development work by us and others has, however, focused almost entirely on the design and evaluation of slide-based planar solid supports for antibody microarrays [5,7,8,9,10,11,12]. Albeit successful, resulting in high-performing antibody microarray set-ups, e.g., [5,16], the potential of (novel) solid surfaces has not been (re-)evaluated in recent years. Additionally and most importantly, a comprehensive view, addressing all of the above surface-related issues in a combined manner, remains to be presented, leaving room for additional array technology platform development and further fostering of our understanding of the antibody-surface interplay.

Ideally, the source plate should be inert and consistently display low protein-binding properties, but in the context of antibody microarrays, published data demonstrating this key feature is absent. In fact, we have recently generated data questioning the printing performances due to significant and inconsistent antibody binding properties displayed by a common and frequently-used source plate. In contrast, the solid microarray surfaces should display high antibody binding capacity and biocompatibility, while any non-specific (background) binding should be minimized [5,7,8,9,10,11]. In our efforts, a hydrophilic polymer slide, to which the antibodies are randomly adsorbed, have so far served as the best-performing solid support [5]. In fact, hydrophilic surfaces have often been reported as favorable for protein adsorption, as they yield homogeneous spots and stable arrays that can be stored for several months [8,17]. Recently, the possibility to use also enzyme-linked immunosorbent assay (ELISA) plates with flat bottoms as solid microarray supports was demonstrated [18,19], representing an attractive assay format more compatible with high-throughput clinical efforts. Notably, the sensing (scanner availability and performance) and compatibility of such well-based array layouts with, in particular, recombinant antibody microarray set-ups remain to be demonstrated.

In this study, we have taken on the first comprehensive view and investigated the combined impact of the solid surfaces on the printing performances, assay format, assay performances, assay processing and sensing of recombinant antibody microarrays. A schematic layout of the array set-up and the technical features that have been addressed are shown in Figure S1. To this end, we have evaluated the use of eight source plates and fourteen solid microarray supports, including both slide- and well-based designs. In parallel, we have also compared manual and semi-automatic array handling, as well as three slide- and/or plate-based scanners for sensing. The results showed that the impact of the solid surfaces is significant and that the overall microarray technology platform performances could be further improved by considering these fundamental phenomena. The observed interplay between the antibodies and the solid surfaces of both the source plate and microarray support, and its impact on array performances in general, is discussed.

## 2. Materials and Methods 

### 2.1. Surfaces

Eight 384-well plates were evaluated as source plates, including clear polypropylene (PP) ABgene natural V-wells (ABgene, Epsom, UK), Corning clear polystyrene (PS) non-binding surface (NBS) treated (Corning, NY, USA), Corning white PS NBS treated (Corning), Genetix clear PS (Molecular Devices, Sunnyvale, CA, USA), Genetic clear PP (Molecular Devices), NUNC clear PS (NUNC, Roskilde, Denmark), NUNC black PP (NUNC) and PerkinElmer PS black ProxiPlate (PerkinElmer Life & Analytical Sciences, Wellesley, MA, USA) (Table 1).

Eleven planar slides and three 96-well plates displaying a range of surface chemistries, surface geometries and binding chemistries were evaluated as antibody microarray solid support (Table 2). The slides were supplied by Corning, Schott (Jena, Germany), PolyAn (Berlin, Germany), Whatman (Florham Park, NY, USA), Arrayit (Sunnyvale, CA, USA), NUNC and Sigma (St Louise, MO, USA). The 96-well plates were supplied by Scienion (Berlin, Germany), Costar and NUNC. Clear MaxiSorp slides were used as a reference when evaluating plate-based supports.

The surfaces were selected to represent a set of potentially high-performing surfaces for antibody microarrays, based on different surface properties and surface chemistries and provided by different vendors.

### 2.2. Antibodies

In total, 102 recombinant single-chain fragment variable (scFv) antibodies (Table S1), stringently selected from an in-house-designed phage display library, were produced in 100 mL *Escherichia coli* cultures. In brief, the antibodies were purified from the cell supernatant using affinity chromatography on Ni^2+^-NTA agarose (Qiagen, Hilden, Germany) and eluted in 250 mM imidazole. The buffer was changed to PBS by extensive dialysis, and the antibodies were stored at 4 °C until used for microarray production. The protein concentration was determined by measuring the absorbance at 280 nm, and the degree of purity and integrity of the scFv antibodies was verified with 10% SDS-PAGE (Invitrogen, Carlsbad, CA, USA).

### 2.3. Samples

Four well-characterized, de-identified human serum samples were used as model samples, including NS80 (a large pool of healthy controls), C1qD (C1q and properdin deficient), C3D (C3 deficient) and C4D (C4 deficient). While the former (healthy) sample was used for a majority of the experiments, the latter three were only used in experiments evaluating antibody specificities. All samples were collected at Skåne University Hospital (Lund, Sweden). Crude serum samples were diluted 1:45 in PBS and labelled with 0.6 mM biotin (EZ-link Sulfo-NHS-Biotin, Pierce, Rockford, IL, USA) for 2 h on ice, as previously described [4,5]. Unconjugated biotin was removed by extensive dialysis against PBS, whereafter the samples were aliquoted and stored at −20 °C. When used for microarray analysis, the labelled samples were diluted 2.5–160 times (10 times in the standard assay) in 1% (*w*/*v*) milk (Semper, Sundbyberg, Sweden) and 1% (*v*/*v*) Tween-20 (Merck, Whitehouse Station, NJ, USA) in PBS (PBS-MT).

Pure BSA (SaveenWerner, Malmö, Sweden) and C3 (Quidel, San Diego, CA, USA) were biotinylated at a molar ratio of biotin:protein of 3:1 as described above. Non-specific surface binding was evaluated by applying 1 μg/mL Alexa-647-labelled streptavidin (SA647) (Invitrogen) or 0.2 mg/mL biotinylated BSA. Biotinylated C3 (5 μg/mL) was used in well-based array experiments.

### 2.4. Microarray Production and Processing

Microarrays were produced by printing 12–60 different scFvs antibodies (at a protein concentration of (40–400 μg/mL) per array, using a non-contact printer based on piezo technology (SciFLEXARRAYER S11, Scienion). Biotinylated BSA (b-BSA) was used as a position marker. Each individual scFv antibody was printed as single droplets (~300 pL/drop) in four to eight replicates at a spot-to-spot distance of 200 or 300 μm. The arrays were stored overnight at room temperature (RT) prior to use. In the long-term storage experiments, the printed antibody microarray slides were stored for up to 42 days prior to analysis.

For the evaluation of source plates, b-BSA (5 μg/mL) from 6–12 different wells was printed in 20 spot replicates on 1–14 individual subarrays. The source plates were incubated 1.5 h prior to printing.

The antibody microarray slides were processed either manually or semi-automatically in a Protein Array Workstation (PAW) (PerkinElmer). For manual processing, individual sub-arrays were created by using silicon incubation chambers (Schott) or a hydrophobic pen (Dako, Glostrup, Denmark) for slides that did not fit the incubation chambers (Silane-Prep (Sigma) and GAPSII (Corning)). Arrays were blocked for 1 h at RT. While well-based arrays were only blocked with 1% (*w*/*w*) milk, 1% (*v*/*v*) Tween-20 in PBS (PBS-MT), several different types of blocking agents (SaveenWerner, BDH Chemicals (Poole, Great Britain), Semper, Merck and Sigma) were evaluated for slide-based solid supports (Table S2). After blocking, the arrays were processed at RT (4 °C for Protein array Workstation (PAW) processing). The slides were first washed in 0.05% (*v*/*v*) Tween-20 in PBS (PBS-T) (0.5% (*v*/*v*) Tween-20 in PBS for PAW processing) before the labelled sample was added for 1 h. After washing in PBS-T, the arrays were incubated for 1 h with 1 μg/mL SA647. Finally, the slides were washed in PBS-T and immediately dried under a stream of nitrogen gas (slides) or in air (plates) and subsequently scanned.

The slides were scanned using a confocal microarray scanner (ScanArrayExpress HT, PerkinElmer,) at three to five different settings of photomultiplier tube (PMT) gain and laser power (LP) and quantified using the ScanArray Express software 4.0 (PerkinElmer) with the fixed circle method. Plates were scanned in an light-emitting diode/charge-couple device (LED/CCD) plate scanner (Sensovation, Radolfzell, Germany), with adjustable LED power and exposure time settings, and automatically quantified in the built-in Genomica software (Genomica, Madrid, Spain). In one experiment, plates and slides were scanned in the confocal LS reloaded combined plate and slide laser scanner (Tecan, Männedorf, Switzerland), with different gain settings (fixed laser power). In this particular case, the plates had to be scanned upside down through the bottom polymer layer due to laser angle restrictions, while slides were scanned with arrays facing upwards.

For each spot, the local background was subtracted. In the case of more than four printed replicate spots, the highest and lowest replicates were first automatically excluded in order to compensate for any local defects, so that each data point used represents the mean value of four replicates. The intensity is given as mean intensity per pixel. For the evaluation of storage stability, arrays were normalized between days of analysis, using a semi-global normalization approach, as previously described [20,21].

It should be noted that in no case was signal overlap from neighboring wells observed when measuring the fluorescence from different wells.

### 2.5. Antibody Footprint

The Web Antibody Modelling procedure [22] was used to generate structural homology models for five of our scFv antibodies in order to estimate the size of their foot print. The mean foot print was estimated to be 50 × 40 Å.

## 3. Results

### 3.1. Evaluation of Source Plates

**Figure 1:**
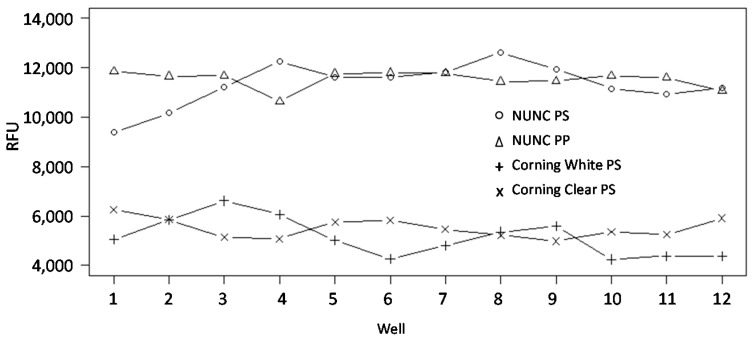
Evaluation of 384-well plates as protein (antibody) source plates for the production of antibody microarrays. The same stock solution of biotinylated BSA was loaded into 12 wells on each source plate and printed on black Maxisorp slides (six subarrays/slide). The spot signal intensities were determined, and the mean value over all six subarrays per well uptake was plotted for the source plates for which the two highest and two lowest signals were obtained.

Eight different 384-well plates were evaluated as a protein (antibody) source plate by printing the same stock solution of biotinylated BSA picked from multiple wells (*n* = 12) on each plate and subsequently determining the signal intensity of the deposited spots (Table 1). The results showed that the reproducibility, expressed as the coefficient of variation (CV), of the printing process decreased in the order of NUNC black PP < Genetix PS < Genetix PP < ABgene PP < Corning clear PS < NUNC clear PS < PerkinElmer < Corning white PS and ranged from 3%–16%. Furthermore, the maximum percentage difference in signal intensity between spots ranged from 11%–55%, again with the NUNC black PP, Genetix PS and Genetix PP plates displaying the smallest variations (Table 1). Hence, the data showed large well-to-well variations in protein binding for some of the source plates, indicating significant surface heterogeneity. Noteworthy, the data also showed that observed spot signal intensities differed (up to 100%) depending on which source plate the BSA was picked from, demonstrating large differences in unwanted protein binding (Figure 1). The highest signal intensities (*i.e.*, the lowest protein binding) were observed when BSA was collected from NUNC PS/NUNC PP and the lowest intensities (*i.e.*, the highest protein binding) from Corning white PS/Corning clear PS plates. The observed protein binding behavior could neither be explained by the nature of the surfaces (e.g., PP *vs.* PS) (Table 1 and Figure 1) nor by the performances of the printer and/or the solid support on which the protein was dispensed (data not shown). Taken together, the data showed that the NUNC black PP plate was the preferred choice as the source plate, while many of the other source plates displayed significant and inconsistent protein binding properties.

### 3.2. Slide-Based Solid Supports: Surface Fouling

**Figure 2:**
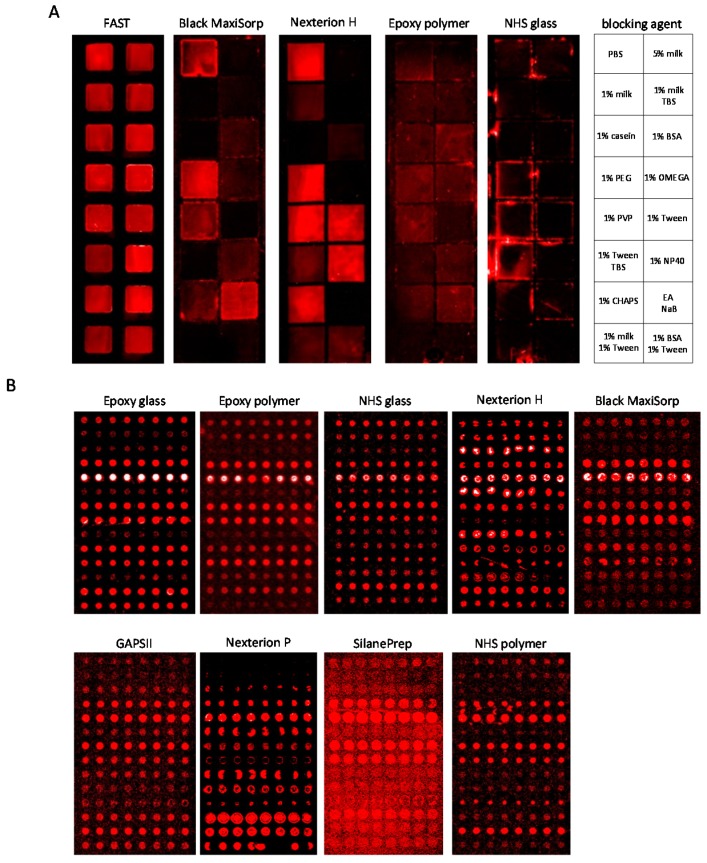
Evaluation of slide-based solid supports for antibody microarrays. The supports were compared with respect to blocking buffer, surface fouling, spot morphology and signal strength. (**A**) The slides (no antibodies printed) were first blocked and then incubated with crude, labelled serum and scanned after washing. Any observed signal intensity represents non-specific background binding. Representative scans of FAST, black MaxiSorp, Nexterion H, epoxy polymer and NHS glass slides are shown. The blocking agents tested are shown to the right; (**B**) Scanned microarray images of 14 × 8 antibody microarrays on epoxy glass, epoxy polymer, NHS glass, Nexterion H, black MaxiSorp, GAPSII, Nexterion P, Silane-Prep and NHS polymer slides. The printed antibody arrays were blocked and then incubated with crude, labelled serum before scanning.

The ability to block the slide-based solid supports from non-specific background binding, *i.e.*, surface fouling, was then examined. To this end, the eleven supports were treated with twenty different blocking solutions (Table S2), and the level of non-specific protein binding to the surface after exposure to a crude, labelled serum sample was determined. Representative results illustrated for five slides and sixteen blocking agents are shown in Figure 2A. The result showed that two (FAST and SuperProtein) of the supports could not be adequately blocked to be compatible with the scanner parameters (laser power and PMT gain) required for the sensitive detection of the recombinant scFv microarrays, and they were therefore excluded from further testing (Figure 2A and Figure S2). In contrast, four supports (black MaxiSorp, Nexterion H, Nexterion P and GAPSII) could be satisfactorily blocked using a few selected blocking agents, while five supports (epoxy polymer, epoxy glass, NHS glass, NHS polymer and Silane-Prep) were efficiently blocked irrespective of the blocking solution used (Figure 2A and Figure S2). Hence, out of eleven supports, nine could be effectively blocked from surface fouling and were selected for further testing.

### 3.3. Slide-Based Solid Supports: Surface Fouling and Spot Properties

Next, the remaining nine solid supports were further compared with respect to surface fouling and spot properties (size, morphology and intensity). Focused antibody microarrays were printed, and the slides were blocked with the top four to six blocking agents identified for each support (Figure S2); a crude, directly-labelled serum sample was analyzed.

Antibody spots with the highest specific signal intensities (range 1400–30,900 Relative Fluorescsnce Unit (RFU), background corrected) were obtained when the solid supports were blocked with 5% (*w*/*v*) BSA (Silane-Prep, Nexterion P and GAPSII), 1% (*w*/*v*) fat-free milk (Nexterion H, NHS polymer and black MaxiSorp) or 0.5% (*v*/*v*) Tween-20 (epoxy glass, epoxy polymer and NHS glass) (Table 3 and Figure 2B). The surface fouling was found to be low (range 150–1100 RFU) and increased in the order of Nexterion P < Nexterion H < epoxy polymer < NHS polymer < NHS glass < black MaxiSorp < Silane-Prep < epoxy glass < GAPSII. However, the surface fouling varied across some of the slides, indicating adverse surface irregularities, a key feature that increased in the order of black MaxiSorp < Nexterion P < epoxy polymer < Nexterion H < NHS polymer < NHS glass < epoxy glass < GAPSII < Silane-Prep (Table 3).

The specific antibody binding pattern also varied considerably across the supports (Figure 2B and Figure S3), demonstrating the impact of the solid support on the antibody array assay. The results showed that the average antibody signal intensity decreased in the order of epoxy glass > epoxy polymer > NHS glass > Nexterion H > black MaxiSorp > GAPSII > Nexterion P > Silane-Prep > NHS polymer (Table 3).

Further, the spot size was uniform (varied ≤1.1-times) on five of nine supports, but differed (≤2.8-times) on all four NHS-functionalized supports (Nexterion H, Nexterion P, NHS glass and NHS polymer) (Figure 2B and Table 3). As could be expected, the average spot size differed across the supports (90–165 μm), indicating different wetting properties. Moreover, circular and homogeneous spots were in general observed on all supports, with the exception of Nexterion P and Nexterion H, which predominantly displayed poor, non-uniform spot morphologies (Table 3). The latter two slides also showed ring-shaped spot drying effects, as did Silane-Prep, GAPSII and epoxy glass slides (Figure 2B).

Taken together, the supports were ranked based on surface fouling (surface regularity), spot intensity, spot size and spot morphology, resulting in three of nine slides being selected for further testing, including epoxy glass, epoxy polymer and black MaxiSorp.

### 3.4. Slide-Based Solid Supports: Reproducibility, Sensitivity and Dynamic Range

The assay reproducibility was determined by analyzing the same serum sample on four identical antibody subarrays printed on two slides of each solid support. The results showed that the spot-to-spot, array-to-array and slide-to-slide reproducibility all decreased in the order of black MaxiSorp > epoxy polymer > epoxy glass (Table 4). Hence, the data implied that the black MaxiSorp slides displayed a more consistent surface well adapted for microarray analysis.

Next, the assay sensitivity was investigated by analyzing seven serial dilutions of a serum sample, with a total protein concentration ranging from 12.5–800 μg/mL, on a focused antibody microarray. The results showed that even low-abundant analytes (e.g., IFN-γ, IL-12 and IL-4) could be detected in the most diluted sample on black Maxisorp and epoxy polymer slides, while a four-times (IFN-γ and IL-12) or eight-times (IL-4) higher total protein concentration was required for detection on the epoxy glass slides (Figure S4). However, the observed signal intensities were stronger on the two epoxy supports than on the black MaxiSorp slides, but on the epoxy supports, the signals were also more saturated at a lower protein concentration, indicating a more limited working dynamic range. Overall, the data implied that the performance of the supports decreased in the order of black MaxiSorp > epoxy polymer > epoxy glass.

### 3.5. Slide-Based Solid Supports: Specificity and Stability

**Figure 3:**
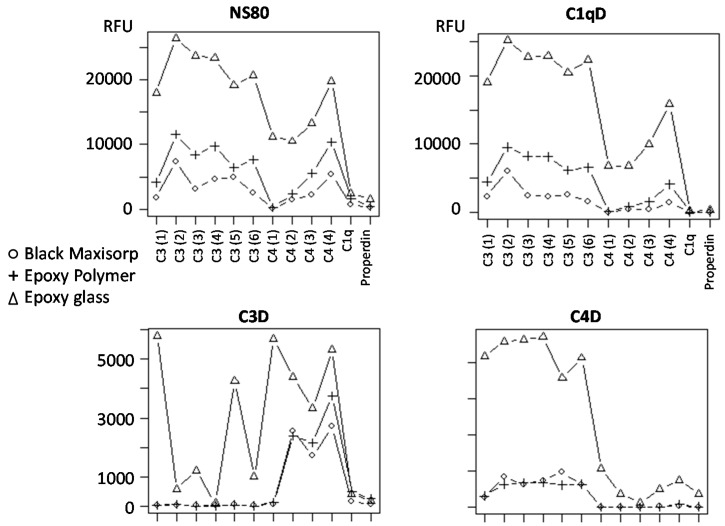
Influence of the slide-based solid support on the specificity of the arrayed antibodies. A 12-plex antibody microarray (6 × C3, 4 × C4, 1 × C1q and 1 × properdin) was used to profile four well-characterized serum samples, including NS80 (large pool from healthy donors), C1qD (C1q and properdin deficient), C3D (C3 deficient) and C4D (C4 deficient).

To investigate the influence of the solid support on the assay specificity, four well-characterized serum samples were profiled, including NS80 (a pool of healthy controls), C1qD (C1q and properdin deficient), C3D (C3 deficient) and C4D (C4 deficient) (Figure 3). As expected, no signals were detected for either C1q/properdin, C3 or C4 when the corresponding deficient serum samples were profiled on black MaxiSorp and epoxy polymer slides. While similar results were observed for the C1qD sample on epoxy glass support, five of six C3-specific antibodies and three of four C4-specific antibodies generated significant signals when profiling the C3D and C4D samples, respectively. Of note, no signals were observed on either of the supports if the antibody microarrays were hybridized with only biotinylated BSA and Alexa-647-labelled streptavidin (SA647) or only SA647 (data not shown). Thus, the results implied that the antibody specificity was maintained on the black Maxisorp and epoxy polymer supports, while the antibody-surface interplay in part impaired the specificity on the epoxy glass support.

The long-term stability of the printed arrays was then tested by printing all arrays on Day 0 and then storing them in a dried out state for 0, 3, 10 and 42 days prior to assay processing. The results showed that the activity of the arrayed antibodies increased with storage time up to ten days on all three supports, whereafter the activity levelled off (Figure S5). The observed increase in antibody activity (signal intensities) was larger on the epoxy glass support than on the other two supports, but this support also gave rise to more non-specific binding to the printed antibodies (*cf.* Figure 3 and Figure S5).

### 3.6. Slide-Based Solid Supports: Semi-Automatic Array Handling

**Figure 4:**
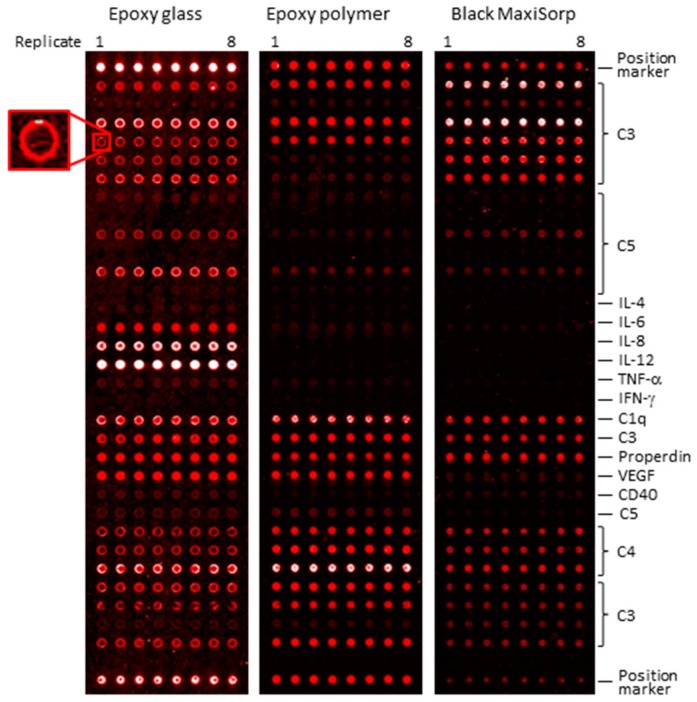
Effect of semi-automatic array handling on slide-based array performance. A serum sample was profiled on a 34 × 8 antibody microarray printed on epoxy glass, epoxy polymer and black MaxiSorp slides that were semi-automatically processed in a Protein Array Workstation. All printed antibodies (*n* = 34) were unique, but several of the clones targeted the same protein (but most likely different epitopes).

Next, we turned from manual to semi-automatic array handling by processing the slides in a Protein Array Workstation (PAW). First, titration of a labelled serum sample showed that a total protein concentration of 1.0 mg/mL (0.2 mg/mL in manual handling) was the preferred concentration to enable also low-abundant analytes to be targeted (data not shown). Second, the spot properties were found to differ considerably across the three supports (Figure 4). While small, homogeneous spots of adequate morphology were observed on both black MaxiSorp and epoxy polymer supports, larger and often ring-shaped spots were obtained on epoxy glass supports (not observed during manual handling, *cf.* Figure 2 and Figure 4). Hence, the black MaxiSorp and epoxy polymer supports, but not epoxy glass, were compatible with automated PAW analysis.

Third, the binding patterns were again found to differ depending on the solid support (*cf.* Figure 4 and Figure S3), demonstrating the importance of the antibody-surface interplay on the antibody activity. While more similar binding patterns were observed on the black MaxiSorp and epoxy polymer slides, the support identified to generate the most non-specific spots, epoxy glass (Figure 3), was also found to generate more apparent detectable spot features. Overall, the performances of the supports were found to decrease in the order of black MaxiSorp > epoxy polymer > epoxy glass.

### 3.7. Well-Based Solid Supports: Surface and LED/CCD Plate Scanner

**Figure 5:**
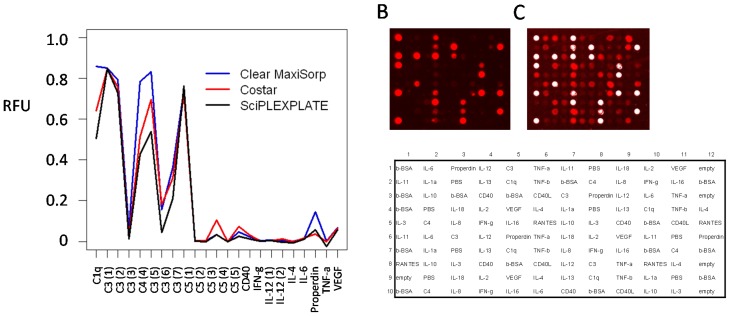
Evaluation of 96-well plates as support for 23-plex antibody microarrays interfaced with an LED/CCD plate scanner. (**A**) Signal intensities from a well-based microarray targeting high- and low-abundant serum proteins on Clear MaxiSorp, Costar and SciPLEXPLATE supports. The data were automatically quantified and processed to a 0–1 scale in an integrated scanner software; (**B**) Microarray image of serum profiling on a well-based antibody microarray targeting predominantly low-abundant analytes, produced in clear MaxiSorp plates and scanned in an LED/CCD scanner (90% LED power, 1200-ms exposure); (**C**) Microarray image of a slide-based antibody microarray (identical to that in (**B**)) produced on black MaxiSorp slides and scanned in a confocal microarray slide laser scanner (80% PMT gain/80% LP). Aliquots of the same biotinylated serum samples were used in both (**B**) and (**C**). The array layout used in (**B**) and (**C**) is shown below the scan images.

Three 96-well plates, SciPLEXPLATE, Costar and clear MaxiSorp, were evaluated as well-based solid supports. To this end, 23-plex antibody microarrays were printed and applied for serum protein profiling, using an LED/CCD-based plate scanner for sensing. The results showed that adequate spot morphology, low surface fouling and similar binding patterns were obtained on all three supports (Figure 5A). Still, the clear MaxiSorp plates generated higher signal intensities for at least a subset of antibodies (e.g., C1q, C3 and properdin), and this plate was therefore selected as the well-based solid support for the remaining part of the study.

Next, we compared the performance of well-based arrays on clear MaxiSorp plates (LED/CCD plate scanner) with that of slide-based arrays on black MaxiSorp slides (confocal slide scanner, denoted PerkinElmer (PE) scanner). The results showed that similar signal intensities were obtained targeting high- to medium-abundant serum analytes (e.g., complement proteins), indicating equal performance (data not shown). However, when instead, predominantly low-abundant serum analytes (e.g., cytokines) were targeted, the observed binding patterns differed, with less analytes being detected on well-based arrays (Figure 5B) than on planar arrays (Figure 5C). Hence, the data indicated that the assay sensitivity was higher for the slide-based array set-up than for the well-based array set-up.

### 3.8. Well-Based Solid Supports: Confocal LS Slide/Plate Scanner

**Figure 6:**
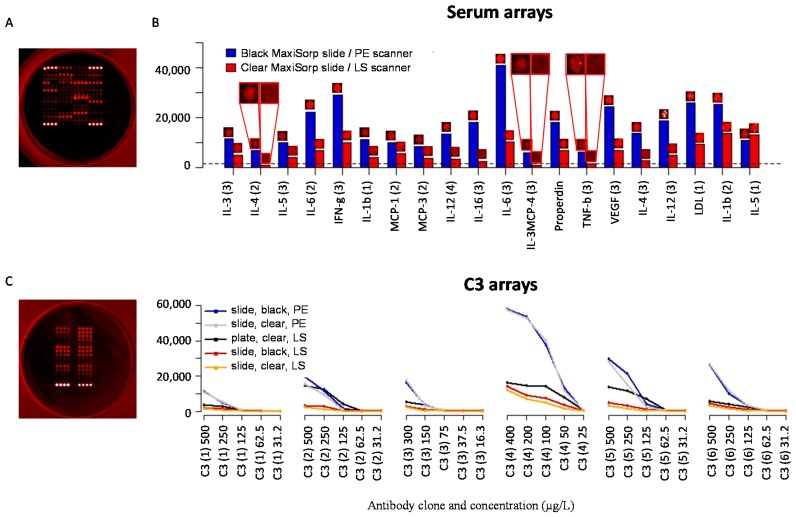
Evaluation of the solid support and scanner on antibody microarray performance. Antibody microarrays, based on 20 antibodies, were printed on slides (clear MaxiSorp and black MaxiSorp) and plates (clear MaxiSorp) and scanned using confocal laser scanners (PE slide scanner and LS plate/slide scanner). (**A**) Microarray image of a 20-plex antibody well-based (clear MaxiSorp) microarray, hybridized with labeled serum and scanned at 120% gain; (**B**) Comparison of signal intensities and spot images (20 antibodies) for well-based arrays (clear MaxiSorp plates, LS scanner) and slide-based arrays (black MaxiSorp slides, PE scanner). Quantified signals were collected from the highest scanning intensity settings used still generating non-saturated spots (180% gain in the LS scanner and 90% PMT gain/90% LP in the PE scanner). The dashed line represents the lower limit of detection (local background plus three standard deviations) for the LS scanner; (**C**) Array of six different C3 antibodies in five dilutions printed in a clear MaxiSorp plate, hybridized with pure, labelled C3 (5 μg/mL) and scanned at 120% gain (LS scanner); (**D**) Comparison of signal intensities from the same C3 assay as in (**C**), run on well-based arrays (clear MaxiSorp plates, LS scanner) and slide-based arrays (black or clear MaxiSorp slides and LS or PE scanner). The quantified signals were taken from scans with the highest possible intensity setting without any spot saturation (60% PMT gain/90% LP for the PE scanner, 144% gain for the slides and 120% gain for the plate in the LS scanner).

In an attempt to increase the sensitivity for the well-based arrays, we introduced a confocal LS slide/plate scanner (denoted LS scanner). Consequently, we repeated the above experiments and compared the performance of well-based antibody arrays (clear MaxiSorp plates, LS scanner) (Figure 6A) with that of slide-based antibody arrays (black MaxiSorp slides, PE scanner) for serum protein profiling focusing on twenty mainly low-abundant analytes. While all but three analytes (IL-4, MCP-4 and TNF-β) now were detected on both set-ups, the results showed that higher and more dynamic signal intensities were still obtained on slide-based arrays (Figure 6B).

Finally, we investigated whether the observed differences in performance were due to the support (clear MaxiSorp *vs.* black MaxiSorp) and/or scanner (PE scanner *vs.* LS scanner). To this end, well-based arrays (clear MaxiSorp) (Figure 6C) and slide-based arrays (clear MaxiSorp and black MaxiSorp), based on serial dilutions of six C3-specific antibodies, were produced and probed with pure, labelled C3 and scanned in the LS scanner and/or PE scanner. First, the results showed that higher and more dynamic signal intensities were obtained when slide-based arrays were scanned using the PE scanner compared to the LS scanner (Figure 6D). Second, similar signal intensities were obtained for slide-based arrays on clear and black MaxiSorp slides, irrespective of the scanner used. In this context, it might be of interest to note that the surface fouling was somewhat higher on clear MaxiSorp than on black MaxiSorp slides, often resulting in lower signal-to-noise ratios (data not shown). Noteworthy, when the same scanner (LS scanner) was used, equal or higher signal intensities were obtained for well-based arrays than for slide-based arrays produced on the same surface (clear MaxiSorp) (Figure 6D). Taken together, the results suggested that the observed differences in performance mainly reflected the choice of scanner. Still, slide-based arrays based on the black MaxiSorp support scanned in the PE scanner appeared to represent the best performing antibody microarray set-up.

## 4. Discussion

To design high-performing antibody microarrays, it will be absolutely necessary to develop an integrated technology platform that has been optimized in all processing steps, where the interplay between the surfaces and the antibody is anticipated to play a central role [9,14]. Despite major efforts, several limitations in the development process have been pin-pointed, leaving room for additional technical improvements and further fostering of our understanding of the antibody-surface interplay. First, very few studies have been reported taking on such a comprehensive view; for a review, see [6,14,23]. Second, in previous studies, a major focus has been placed on the choice of solid array support, while the impact of the source plate has not been reported [14]. Third, the potential of (novel) array surfaces and array/assay formats has not been evaluated or re-evaluated in recent years (to explore potential cooperative effects due to other parallel developments of the array technology). Fourth, the sensing (scanner performance) and compatibility of well-based array layouts with in particular recombinant antibody microarrays are features that remain to be explored and exploited. In this study, we have therefore re-visited and further investigated the antibody-surface interplay, including both source plates and array surfaces. Uniquely, we took on a comprehensive approach and demonstrated the impact of the antibody-surface interplay on each step of the microarray process, including the printing process, array/assay performance (e.g., functionality, sensitivity and stability), degree of automation, array/assay format and sensing.

It was demonstrated that the source plate had a profound influence on the printing performance, as reflected by significant and inconsistent protein binding, indicating unexpectedly high protein binding properties and adverse well-to-well surface irregularities. Hence, the actual printing concentration of an antibody differed not only from plate-to-plate, but also from well-to-well of the same plate, in the end impairing the overall assay reproducibility and sensitivity. The hydrophobicity of the surfaces is an important feature when discussing non-specific protein loss due to adsorption [24]. Although the protein loss could not simply be explained by the nature of the surfaces (PP *vs.* PS, PS being more hydrophobic in general), the highest protein loss was indeed observed on two PS surfaces. This lack of correlation might to some extent be explained by the fact that the surfaces were subjected to various surface treatments during their production process, which might influence their protein loading capacity in different ways. Hence, further experiments will be required to unravel their protein binding behavior. To the best of our knowledge, published data addressing this fundamental choice of source plate for antibody (protein) microarrays is still very limited [14]. Here, we thus present the first validated (inert, low and consistent protein binding properties) source plate, based on black polypropylene, suitable for antibody microarray production.

Turning to the solid array support, we used the ability to block the surfaces for non-specific background binding, or surface fouling, as a key cut-off parameter [5,7,8,9,10,11]. Hence, increasing the signal-to-noise ratio is a vital platform parameter. The blocking step might be problematic in that: (i) large blocking molecules could sterically hinder binding to smaller probes (the molecular weight of our scFv antibodies is 28 kDa); (ii) the blocking molecules might not be completely non-fouling; and (iii) the blocking molecules might interact non-specifically with the sample. In the end, we found protein- (BSA or milk) and/or Tween-20-, but not polymer-based, blocking buffers to be the preferred blocking agents (for the surfaces tested here).

While nine of 11 slides could be adequately passivated, we failed completely to block two supports, FAST slides (nitrocellulose surface with high protein binding capacity [25,26,27] and SuperProtein slides (hydrophobic surface based on a 150 μm-thick polymer membrane). We have, however, repeatedly observed lower sensitivity and higher background binding on nitrocellulose- and polymer matrix-based substrates [5], implying that these surfaces might not be compatible with recombinant scFv arrays targeting crude serum proteomes, but rather seem more suitable for reversed antibody arrays [28,29]. Noteworthy, hydrophobic surfaces have in general also been found to yield a higher degree of denatured immobilized proteins and non-specific binding [30], while we found a hydrophilic polymer support (black MaxiSorp) to be the best performing support in both this and a previous study [5].

Six of the slide-based solid supports displayed uneven background signals (e.g., GAPSII and Silane-Prep) and/or non-homogeneous spots (e.g., GAPSII, Silane-Prep, Nexterion P and Nexterion H) of different sizes (e.g., Nexterion H, Nexterion P, NHS glass and NHS polymer) and intensities (Silane-Prep and NHS polymer), indicating unfavorable surface irregularities impairing the immobilization and/or functionality of the arrayed antibodies. Silanized surfaces (GAPSII and Silane-Prep) are inherently hydrophobic, which might, as discussed above, cause protein to denature [30], while supplementing the printing buffer with glycerol or other additives to protect the antibodies might be beneficial [8,13]. We did not, however, optimize the printing buffer for each individual surface within this study, due to logistical limitations and technical issues (e.g., spotter compatibility with glycerol), but rather aimed to find a support compatible with the standard buffer (PBS) predominantly used in our overall work schedule.

Consequently, three of eleven slide-based supports, including black MaxiSorp, epoxy polymer and epoxy glass, were thus subjected to a more detailed evaluation. While the spot-to-spot reproducibility was excellent [14] on all three supports indicating adequate local surface regularity, the array-to-array and slide-to-slide reproducibility was only adequate on black MaxiSorp, indicating more pronounced surface irregularities across/between slides of the two epoxy supports and, in particular, the epoxy glass slides. In accordance, the ability of black MaxiSorp to act as planar support for highly reproducible antibody array assays have previously been demonstrated [5]. In addition, the two best-performing substrates, black MaxiSorp and epoxy polymer, were both found to enable selected low-abundant serum analytes to be detected in crude, directly biotinylated serum diluted 7200 times, corresponding to a total protein concentration of about 12.5 μg/mL. This assay sensitivity is at the very high end of what have previously been reported for other array set-ups, e.g., [5,16]. The superior behavior of the black MaxiSorp and epoxy polymer supports was further manifested by the fact that these two surfaces, but not epoxy glass, were compatible with the PAW instrument (more stringent washes, *etc.*), enabling semi-automatic array handling.

The protein loading capacity of array surfaces is a key feature, which in direct experimental terms is very difficult to assess. The footprint of our scFv antibodies was estimated to be 50 × 40 Å, suggesting a theoretical loading of 5 × 10^10^ protein molecules per mm^2^, assuming a monolayer. However, as we used surfaces with both a 2D and 3D surface geometry, the relevance of this number could be argued. Although important, we therefore rather used the on-chip functional activity, which can be experimentally determined in terms of intensity per spot, as an array surface ranking tool.

In accordance with previous results [11,12,14,31], the on-chip functionality of the arrayed antibodies appeared to increase with storage time on all three supports and levelled off after ten days. The cause(s) for this is still not clear, but could at least partly be explained by reasoning that those probes that denatured upon deposition onto the solid support have refolded and thereby regained their functional activity. Most importantly, on Day 0, the activity of the arrayed antibodies and the observed binding patterns differed across these three top supports, indicating striking differences in the antibody-surface interplay. Although strong(er) signals in many cases were observed on the epoxy glass support, many of these spots also appeared to be false-positive, indicating unfavorable surface-antibody interactions. Additional experiments will be required in order to elucidate the underlying reasons (e.g., electrostatic and/or hydrophobicity effects) for these key observations. In addition, even though this platform is based on recombinant scFv antibodies constructed around the same identical scaffold, microarray adapted by molecular design [5,14,23], thus exhibiting similar molecular properties (differing only in their complementarity determining regions), we observed antibody clone-dependent differences. In accordance with previously-published work, this highlighted the importance of carefully evaluating the impact of the solid support on the functional on-chip activity/stability of the arrayed antibodies [5,7,8,9,10,11,12] and of using microarray-adapted antibody probes that are as similar as possible to minimize any bias introduced by the probes [23].

Well-based antibody microarrays are an attractive approach, especially from a clinical assay implementation point of view, and the first generation of mainly low-to-medium dense set-ups have been presented [18,19,32,33,34,35]. Here, we designed the very first well-based recombinant scFv antibody microarray set-up. Compared to slide-based solid supports, the availability of plates with specific surface (3D) coatings represents a limitation. While classical ELISA supports, such as hydrophobic polystyrene in some cases, have been found to cause partial protein denaturation [36], we tested three polystyrene-based plates that all resulted in highly functional antibody microarrays, with clear MaxiSorp plates representing the top candidate. Unfortunately, black MaxiSorp plates could not be evaluated due to plate scanner incompatibility.

The overall performance (e.g., sensitivity) of the well-based arrays were found to be adequate, in particular when targeting high- to medium-abundant serum analytes, clearly indicating the potential of the set-up. Compared to the slide-based array set-up, the results first showed lower and less dynamic signal intensities, indicating inherently lower performance and, thus, potential limitations towards targeting low-abundant serum analytes. This scenario was, however, refined, and additional experiments showed that equal or higher signal intensities were obtained for well-based arrays than for slide-based arrays when the key technical differences (choice of surface and scanner) were neutralized, suggesting that the initially observed differences in performance mainly reflected the choice of scanner (and its performance). Of note, the plates had to be scanned upside down in the LS scanner due to laser angle restrictions associated with standard format 96-well plates, while the sensitivity might be even further improved if scanner-compatible plates could be utilized (not pursued due to logistical limitations). Still, the results clearly outlined the potential of well-based recombinant antibody microarrays for protein expression profiling. 

## 5. Conclusions 

In conclusion, we have taken on the first comprehensive view and investigated the impact of the solid surfaces on the printing performance, assay format, assay performance, assay execution and sensing for recombinant antibody microarrays. The results clearly demonstrated the importance of considering the surface-antibody interplay when designing high-performing antibody microarrays. The practical output is two high-end array platforms, based on slide-based arrays (black polystyrene) and well-based arrays (clear polystyrene), paving the way for future protein expression profiling efforts.

## Figures and Tables

**Table 1:** Evaluation of 384-well plates as protein (antibody) source plates for microarray production. The same stock solution of biotinylated bovine serum albumin (BSA) was loaded into 12 wells on each source plate and printed on black MaxiSorp slides (14 subarrays/slide). The spot signal intensities were determined and reported in terms of mean *CV*-value for all spots and the maximum signal intensity difference.

Source Plate	*CV*-Value	Maximum Signal Difference ^(1)^
PP/black/NUNC ^(2)^	3%	11%
PP/clear/Genetix ^(3)^	6%	20%
PP/clear/ABgene ^(4)^	7%	36%
PS/clear/Genetix ^(5)^	5%	19%
PS/clear/NUNC ^(6)^	8%	26%
PS, NBS-treated/clear/Corning ^(7)^	8%	55%
PS/black/PerkinElmer ^(8)^	10%	30%
PS, NBS-treated/white/Corning ^(9)^	16%	55%

^(1)^ ((highest signal intensity—lowest signal intensity)/lowest signal intensity) × 100%; ^(2)^ Stated to display lower binding capacity so that proteins and DNA will not bind, allowing for complete protein recovery; ^(3)^ no additional surface property information at hand; ^(4)^ stated to be a highly polished surface making it low protein binding and chemically inert; ^(5)^ no additional surface property information at hand; ^(6)^ stated to display lower binding capacity so that proteins and DNA will not bind, allowing for complete protein recovery; ^(7)^ stated to be a nonionic hydrophilic surface (polyethylene oxide-like) that minimizes molecular interactions; ^(8)^ no additional surface property information at hand; ^(9)^ Stated to be a nonionic hydrophilic surface (polyethylene oxide-like) that minimizes molecular interactions.

**Table 2:** Slide- and well-based surfaces evaluated as solid supports for antibody microarrays.

Format	Name	Surface Chemistry	Surface Geometry ^a^	Binding Chemistry	Supplier
Slides	Nexterion H	NHS^b^ polymer	3D	Covalent	Schott
	Nexterion P	Hydrophilic NHS polymer	3D	Covalent	Schott
	GAPS II	Aminopropylsilane	2D	Ionic	Corning
	NHS glass	NHS glass	3D	Covalent	PolyAn
	NHS polymer	NHS polymer	3D	Covalent	PolyAn
	Epoxy glass	Epoxy glass	3D	Covalent	PolyAn
	Epoxy polymer	Epoxy polymer	3D	Covalent	PolyAn
	FAST	Nitrocellulose	3D	Adsorption	Whatman
	SuperProtein	Hydrophobic polymer	2D	Adsorption	Arrayit
	Silane-Prep	Aminoalkylsilane glass	2D	Ionic	Sigma
	Black MaxiSorp	Hydrophilic polymer	2D	Adsorption	NUNC
	Clear MaxiSorp*	Hydrophilic polymer	2D	Adsorption	NUNC
96-well plates	Clear MaxiSorp	Hydrophilic polymer	2D	Adsorption	NUNC
	SciPLEXPLATE	Polystyrene	2D	Adsorption	Scienion
	Costar	Polystyrene	2D	Adsorption	Corning

^a^ 3D—three-dimensional, 2D—two-dimensional; ^b^ N-Hydroxysuccinimide; * This slide was only used as a reference when evaluating plate-based surfaces.

**Table 3:** Comparison of the best blocking solution for nine solid supports.

Slide	Blocking Solution	Background Intensity ^(1)^	Background Homogeneity ^(2)^	Spot Intensity ^(3)^	Spot Size (μm)	Spot Morphology ^(4)^
Black MaxiSorp	5% (*w*/*v*) milk PBS	500	+ + +	11,600	125–140	+ +
Epoxy glass	0.5% (*v*/*v*) Tween-20 PBS	800	− −	30,900	130–140	+
Epoxy polymer	0.5% (*v*/*v*) Tween-20 PBS	250	+	14,700	130–150	+ + +
GAPSII	5% (*w*/*v*) BSA PBS	1100	− − −	6300	150–160	+ +
Nexterion H	1% (*w*/*v*) milk TBS	200	+	8900	60–120	− −
Nexterion P	5% (*w*/*v*) BSA PBS	150	+ +	5300	60–170	− − −
NHS glass	0.5% (*v*/*v*) Tween-20 PBS	350	−	13,900	70–160	+
NHS polymer	1% (*w*/*v*) milk TBS	300	−	1400	60–160	+ +
Silane-Prep	1% (*w*/*v*) BSA PBS	700	− − −	3700	160–170	−

^(1)^ The mean intensity (RFU) of the non-specific background binding intensity measured at four randomly-selected positions (70% PMT gain and 70% laser power); ^(2)^ the background homogeneity is graded from best (+ + +) to worst (− − −); ^(3)^ mean spot signal intensity (RFU) for anti-C1q, anti-C3, anti-C4, anti-CD40, anti-IL-8, anti-properdin and anti-VEGF (70% PMT gain and 70% laser power); ^(4)^ the spot morphology is graded from best (+ + +) to worst (− − −).

**Table 4:** Reproducibility of the antibody microarray set-up, expressed as coefficient of variation (CV), for non-normalized data. The same serum sample was analysed on four identical 14 × 8 antibody subarrays printed on two separate slides of each solid support.

Array Features	Epoxy Glass	Epoxy Polymer	Black MaxiSorp
Spot-to-spot	4.7 ± 7.7	4.1 ± 5.2	3.3 ± 3.0
Array-to-array	26.4 ± 15.0	19.5 ± 6.5	3.6 ± 0.6
Slide-to-slide	34.3 ± 11.6	29.0 ± 15.7	12.2 ± 3.6

## Supplementary Materials

The following are available online at http://www.mdpi.com/2076-3905/5/2/16/s1. Figure S1: Schematic illustration of the recombinant antibody microarray technology platform, also highlighting the main technical issues addressed in the study, Figure S2: Evaluation of blocking buffers for slide-based solid supports. The slides were blocked with 16 different blocking solutions (Table S2) and then exposed to crude, directly-biotinylated serum sample, whereafter any non-specific background binding (surface fouling) was detected, Figure S3: Comparison of antibody binding pattern on three different slide-based solid supports. A serum sample was profiled on a 14-plex antibody microarray printed on slide-based supports, including black MaxiSorp, epoxy polymer and epoxy glass. Quantified signals were collected from the highest possible scanner setting without any spot saturation (60% PMT gain/90% LP for black MaxiSorp slides and 50% PMT gain/90% LP for the epoxy-coated slides), Figure S4: Comparison of assay sensitivity and the dynamic range of slide-based antibody microarrays. Serial dilutions of a serum sample, ranging from a total protein concentration of 12.5–800 µg/mL, was profiled on black MaxiSorp, epoxy polymer and epoxy glass, Figure S5: Evaluation of long-term storage on the activity of slide-based antibody microarrays. Arrays were printed on black MaxiSorp, epoxy polymer and epoxy glass slides. All antibody microarrays were printed on Day 0 and then stored dried out for 0, 3, 10 or 42 days prior to array handling (blocking, washing, sample incubation, *etc*.). The antibody activity for four representative antibodies, targeting IL-4, TNF-α, IFN-γ and C1q, was assessed, Table S1: Antibodies used, Table S2: Blocking agents evaluated for blocking of slide-based solid supports.

## Abbreviations

b-BSA: biotin-BSA; EA: ethanolamine; LP: laser power; NaB: sodium borate; NBS: non-binding surface; NC: nitrocellulose; NTA: nitrilotriacetic acid; PAW: Protein Array Workstation; PP: polypropylene; PS: polystyrene; RFU: relative fluorescence units; SA647: streptavidin-Alexa647; scFv: single-chain fragment variable.
