# Supplementary material for: Evaluation of Solid Supports for Slide- and Well-Based Recombinant Antibody Microarrays
Source: Microarrays (Basel). 2016 Jun 8;5(2):16. doi: 10.3390/microarrays5020016 (PMC5003492; doi:10.3390/microarrays5020016)
Supplement: Supplementary file 1 [file microarrays-05-00016-s001.pdf]

Supplementary Material: Evaluation of Solid Supports for Slide- and Well-Based Recombinant Antibody Microarrays

Anna S. Gerdtsson, Linda Dexlin-Mellby, Payam Delfani, Erica Berglund, Carl A. K. Borrebaeck and Christer Wingren

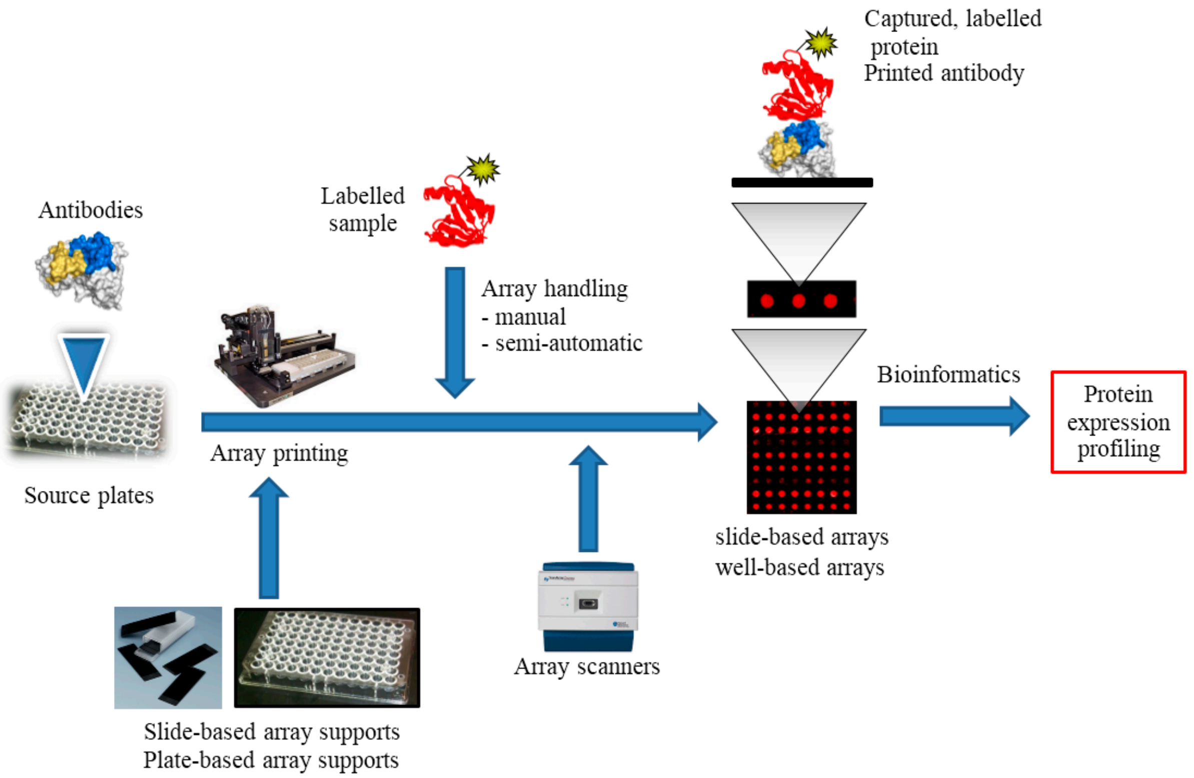

Figure S1. Schematic illustration of the recombinant antibody microarray technology platform, also highlighting the main technical issues addressed in the study.

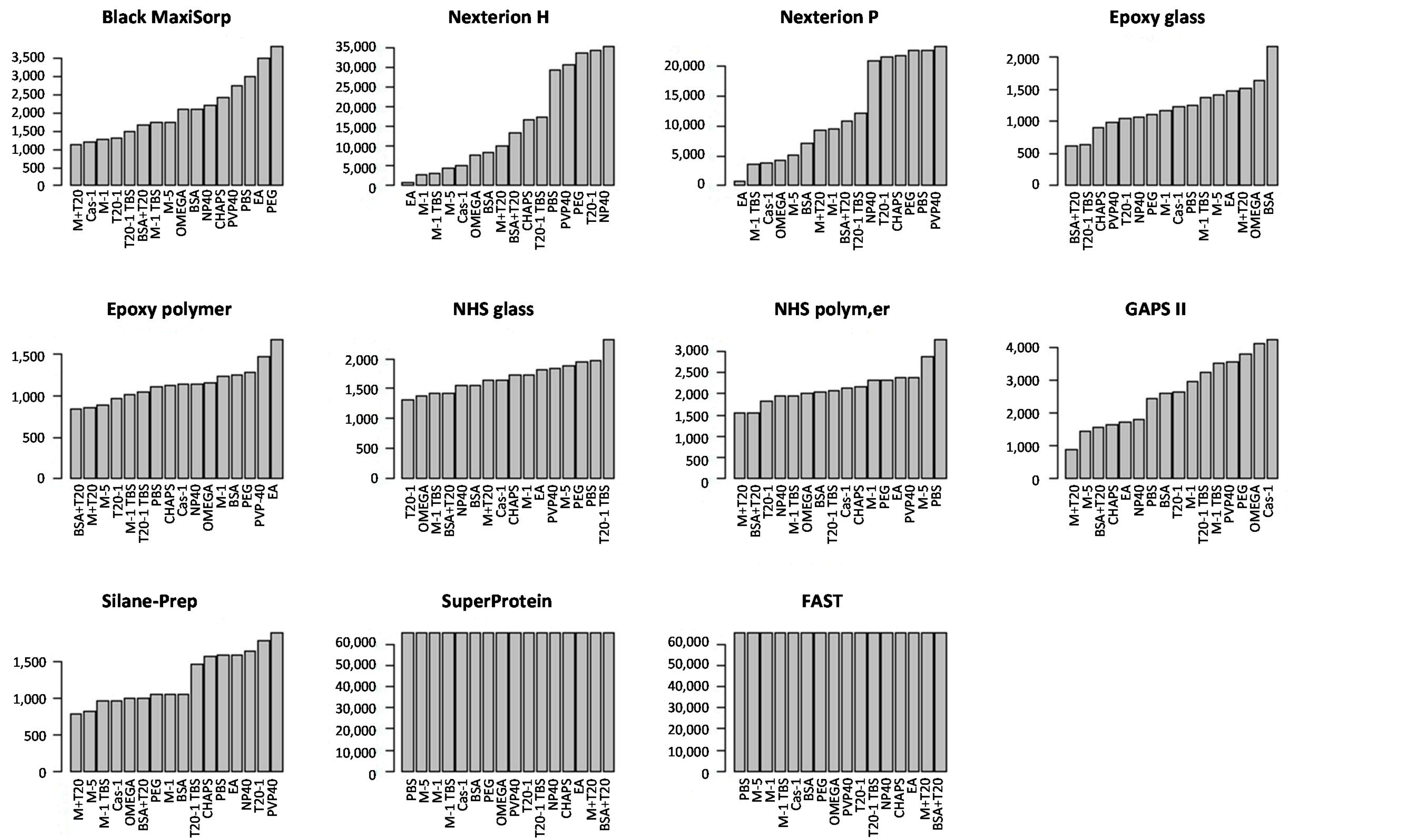

Figure S2. Evaluation of blocking buffers for slide-based solid supports. The slides were blocked with 16 different blocking solutions (Table S2) and then exposed to crude, directly-biotinylated serum sample, whereafter any non-specific background binding (surface fouling) was detected.

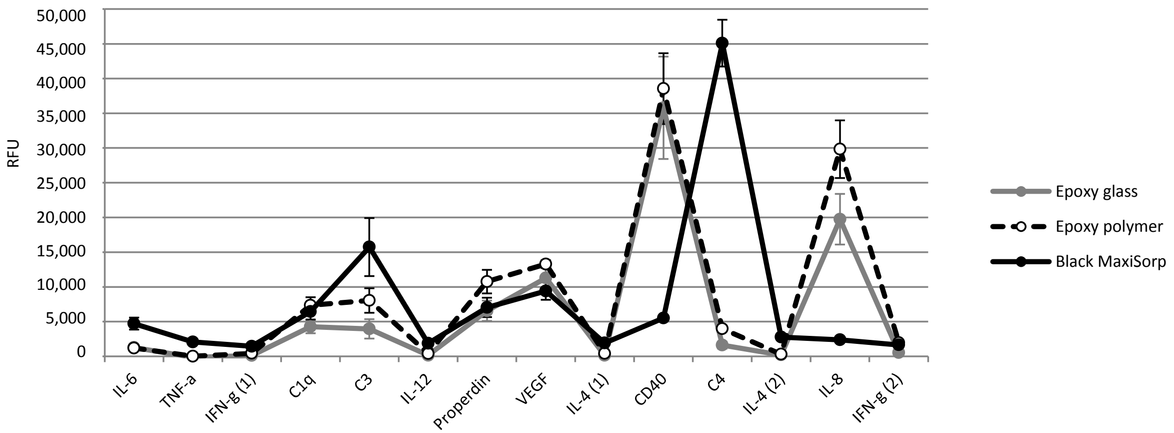

Figure S3. Comparison of antibody binding pattern on three different slide-based solid supports. A serum sample was profiled on a 14-plex antibody microarray printed on slide-based supports, including black MaxiSorp, epoxy polymer and epoxy glass. Quantified signals were collected from the highest possible scanner setting without any spot saturation (60% PMT gain/90% LP for black MaxiSorp slides and 50% PMT gain/90% LP for the epoxy-coated slides).

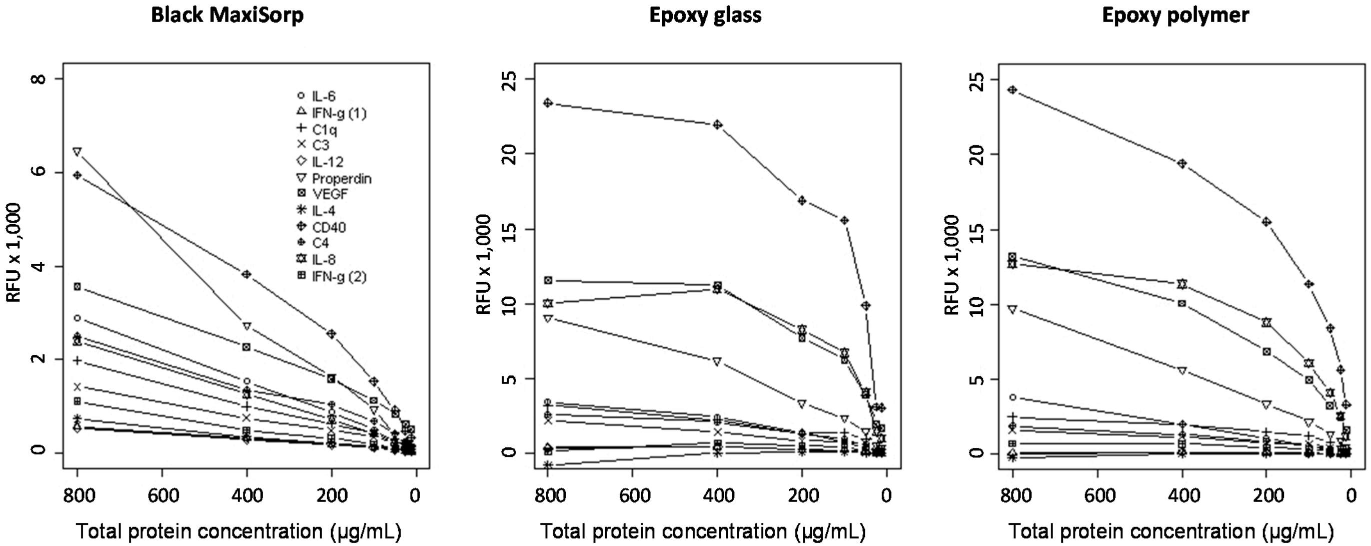

Figure S4. Comparison of assay sensitivity and the dynamic range of slide-based antibody microarrays. Serial dilutions of a serum sample, ranging from a total protein concentration of 12.5–800 µg/mL, was profiled on black MaxiSorp, epoxy polymer and epoxy glass.

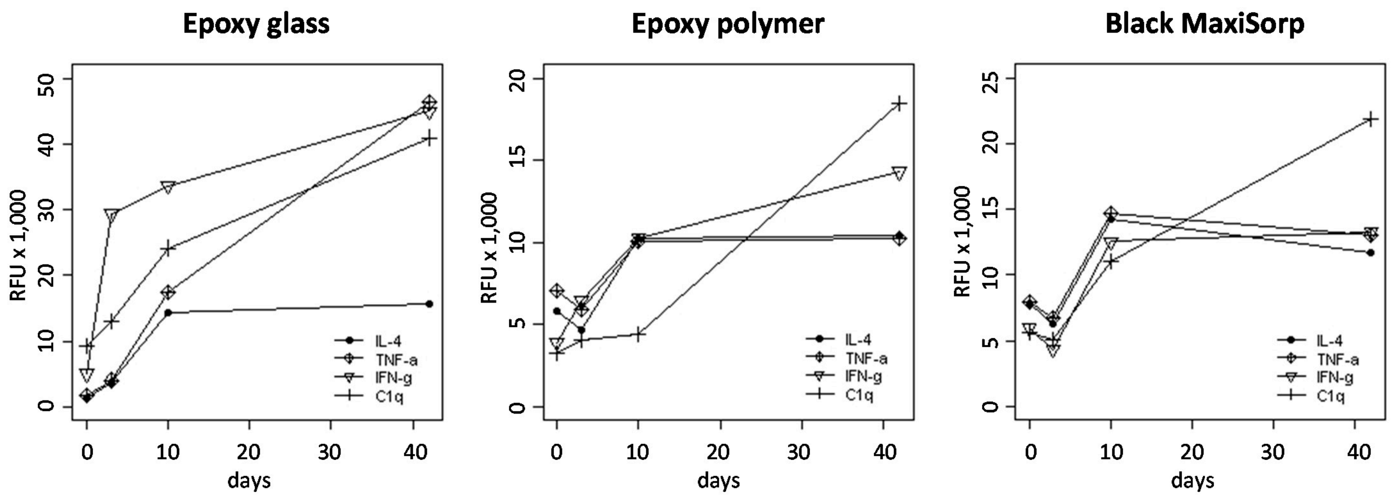

Figure S5. Evaluation of long-term storage on the activity of slide-based antibody microarrays. Arrays were printed on black MaxiSorp, epoxy polymer and epoxy glass slides. All antibody microarrays were printed on Day 0 and then stored dried out for 0, 3, 10 or 42 days prior to array handling (blocking, washing, sample incubation, *etc*.). The antibody activity for four representative antibodies, targeting IL-4, TNF-α, IFN-γ and C1q, was assessed.

Table S1. Antibodies used.

Antigen	No. of Antibody Clones	Antigen	No. of Antibody Clones
Angiomotin	1	IL-9	2
Apolipoprotein A1	1	IL-10	3
Bruton's Tyrosine Kinase	1	IL-11	2
C1 esterase inhibitor	2	IL-12	3
C1q	1	IL-13	3
C1s	1	IL-16	3
C3	12	IL-18	2
C4	4	Integrin α-10	1
C5	9	Integrin α-11	1
CD40	2	LDL	2
CD40 ligand	1	Leptin	1
Cystatin C	1	Lewis x	1
Factor B	2	Lewis y	1
GLP-1R	1	MCP-1	2
GM-CSF	1	MCP-3	1
IFN-γ	2	MCP-4	1
IgM	1	Mucin 1	1
IL-1α	2	Procathepsin	1
IL-1β	2	Properdin	1
IL-1ra	1	RANTES	2
IL-2	1	TGF-β	1
IL-3	3	TM peptide	1
IL-4	3	TNF-α	1
IL-5	2	TNF-β	2
IL-6	3	VEGF	2
IL-8	1		

Table S2. Blocking agents evaluated for blocking of slide-based solid supports.

Name	Blocking Agent	Buffer	Type
PBS	None	PBS	Control
BSA-1	1% (*w*/*v*) BSA	PBS	Protein
BSA-5	5% (*w*/*v*) BSA	PBS	Protein
Cas-1	1% (*w*/*v*) casein	PBS	Protein
Cas-5	5% (*w*/*v*) casein	PBS	Protein
M-1	1% (*w*/*v*) non-fat dry milk	PBS	Protein
M-1 TBS	1% (*w*/*v*) non-fat dry milk	TBS	Protein
M-5	5% (*w*/*v*) non-fat dry milk	PBS	Protein
M-5 TBS	5% (*w*/*v*) non-fat dry milk	TBS	Protein
OMEGA[(a)]	1% (*w*/*v*) OMEGA	PBS	Polymer
PEG	1% (*w*/*v*) PEG2000	PBS	Polymer
PVP40	1% (*w*/*v*) PVP 40	PBS	Polymer
CHAPS[(b)]	1% (*w*/*v*) CHAPS	PBS	Detergent
NP40[(c)]	1% (*v*/*v*) NP-40	PBS	Detergent
T20-1	1% (*v*/*v*) Tween-20	PBS	Detergent

Table S2. *Cont.*

Name	Blocking Agent	Buffer	Type
T20-1 TBS	1% (*v*/*v*)Tween-20	TBS	Detergent
TX100	1% (*v*/*v*) Triton-X-100	PBS	Detergent
BSA + T20	1% (*w*/*v*) BSA + 1% (*v*/*v*) Tween-20	PBS	Mix
M + T20	1% (*w*/*v*) non-fat dry milk + 1% (*v*/*v*) Tween-20	PBS	Mix
EA	25 mM ethanolamine	100 mM sodium borate	Other

(a) OMEGA= *N*-Octanoyl-*N*-methylglucamine; (b) CHAPS= 3-[(3-Cholamidopropyl)dimethylammonio]-1-propanesulfonate hydrate; (c) NP40 = 4-Nonylphenyl poly(ethylene glycol)
